# Lipoprotein(a), family history of cardiovascular disease, and incidence of heart failure

**DOI:** 10.1016/j.jlr.2023.100398

**Published:** 2023-06-03

**Authors:** Hai-Peng Wang, Na Zhang, Yu-Jie Liu, Tian-Long Xia, Guo-Chong Chen, Jing Yang, Fu-Rong Li

**Affiliations:** 1 Department of Cardiology, The First Affiliated Hospital of Soochow University, Suzhou, China; 2 School of Public Health, Shanghai Jiao Tong University School of Medicine, Shanghai, China; 3 Department of Nutrition and Food Hygiene, School of Public Health, Suzhou Medical College of Soochow University, Suzhou, China; 4 Division of Public Health Emergency, Shenzhen Center for Disease Control and Prevention, Shenzhen, Guangdong, China; 5 Department of Clinical Nutrition, The First Affiliated Hospital of Soochow University, Suzhou, China; 6 Shenzhen Key Laboratory of Cardiovascular Health and Precision Medicine, Southern University of Science and Technology, Shenzhen, China; 7 School of Public Health and Emergency Management, Southern University of Science and Technology, Shenzhen, China; 8 Department of Epidemiology, School of Public Health, Southern Medical University, Guangzhou, China

**Keywords:** family history of cardiovascular disease, heart failure, Lipoprotein(a)

## Abstract

Lipoprotein(a) (Lp(a)) is a largely genetically determined biomarker for cardiovascular disease (CVD), while its potential interplay with family history (FHx) of CVD, a measure of both genetic and environmental exposures, remains unclear. We examined the associations of Lp(a) in terms of circulating concentration or polygenetic risk score (PRS), and FHx of CVD with risk of incident heart failure (HF). Included were 299,158 adults from the UK Biobank without known HF and CVD at baseline. Hazards ratios (HRs) and 95% Cls were estimated by Cox regression models adjusted for traditional risk factors defined by the Atherosclerosis Risk in Communities study HF risk score. During the 11.8-year follow-up, 5,502 incidents of HF occurred. Higher levels of circulating Lp(a), Lp(a) PRS, and positive FHx of CVD were associated with higher risks of HF. Compared with individuals who had lower circulating Lp(a) and no FHx, HRs (95% CIs) of HF were 1.36 (1.25, 1.49), 1.31 (1.19, 1.43), and 1.42 (1.22, 1.67) for those with higher Lp(a) and a positive history of CVD for all family members, parents, and siblings, respectively; similar results were observed by using Lp(a) PRS. The risk estimates for HF associated with elevated Lp(a) and positive FHx were attenuated after excluding those with incident myocardial infarction (MI) during follow-up. Lp(a) and FHx of CVD were independent risk factors for incident HF, and the highest risk of HF was observed among individuals with both risk factors. The association may be partly mediated by myocardial infarction.

Cardiovascular disease (CVD) remains the foremost cause of morbidity and mortality on a global scale. While there has been a decline in CVD-related mortality over the past few decades, the prevalence of heart failure (HF) has steadily increased during the same period (1, 2). In the United Kingdom, HF is currently estimated to affect approximately 900,000 individuals, with up to 17% of diagnosed patients dying within the first year of diagnosis (3), highlighting the need for early identification and treatment of patients at risk.

Circulating Lp(a) is largely determined by single-nucleotide variants (SNVs) at the LPA gene. This biomarker consists of a proatherogenic and a prothrombotic component that has been linked to the pathogenesis of CVD (4, 5). Despite the conclusive role of Lp(a) in atherosclerotic cardiovascular disease (ASCVD), inquiry into its role in HF is limited (6, 7, 8).

It is well established that both genetic and environmental factors may contribute to the development of HF (9, 10). In fact, circulating Lp(a) is a primarily genetically determined risk-enhancing factor, while family history (FHx) of CVD, which reflects both the inherited predisposition to CVD and long-term environmental exposures, is another well-confirmed risk factor for CVD development (11, 12, 13, 14). National guidelines recommend Lp(a) testing among patients with FHx of CVD (15, 16); also, according to the dyslipidemia guidelines issued by the European Society of Cardiology/European Atherosclerosis Society in 2019, it is recommended to contemplate the assessment of Lp(a) levels in individuals with moderate and high ASCVD risk (15). Indeed, FHx of CVD may aggravate the detrimental impact of elevated Lp(a) on CVD risk. This was evidenced by a study of the ARIC (Atherosclerosis Risk In Communities) cohort, which found elevated plasma Lp(a) and positive FHx of CVD to have independent and additive joint associations with ASCVD risk in the general population (17). However, there is limited data documenting the independent and joint effects of Lp(a) and FHx on HF risk and, as such, whether the above recommendations also apply to HF prevention remains to be assessed.

In this study, we used data from the UK Biobank study, a population-based prospective study of approximately 0.5 million participants from different regions across the United Kingdom to assess the associations of circulating Lp(a), Lp(a) polygenetic risk score (PRS), and FHx of CVD and their potential interplays with the incidence of HF and to evaluate the implication of these measures for risk categorization of HF beyond traditional risk factors.

## Materials and Methods

### Study population

The UK Biobank is an ongoing prospective cohort of roughly 0.5 million individuals aged 40–69 years, enrolled from 22 centers across England, Wales, and Scotland during the period of 2006–2010 (18). At the time of enrollment, the UK Biobank gathered diverse measurements from participants and procured extensive information regarding sociodemographic factors, lifestyle, and medical conditions. The study protocol was approved by the North West Multi-Centre Research Ethics Committee and carried out according to the Declaration of Helsinki principles. All participants provided written informed consent. Further details on the UK Biobank are available elsewhere (19).

### Lp(a) measurement and FHx assessment

In the UK Biobank, participants’ blood samples were collected during the baseline assessment visit. Circulating Lp(a) concentrations (nmol/L) were measured using an immunoturbidimetric method on the Beckman Coulter AU5800 platform (Randox Bioscience), which is isoform insensitive (20). Information on various types of first-degree relatives’ medical histories, including the illnesses of the father, mother, and siblings, was collected via questionnaires by trained nurses. Specifically, participants were asked “Has/did your father/mother/ever suffer from? (You can select more than one answer)” and “Have any of your brothers or sisters suffered from any of the following diseases? (You can select more than one answer)”. Those who reported a history of heart disease among the above first-degree relatives were defined as having an FHx of CVD among the very relative. In the present study, parental history of CVD was defined as reporting a positive history of heart disease for either or both parents, while sibling history of CVD was defined as reporting brothers or sisters had heart disease. Accordingly, all FHx was defined by combining the CVD history of both parents and siblings.

### Polygenetic risk score for Lp(a)

Circulating Lp(a) levels are largely determined by genetics; as such, genotyping array data from participants of the UK Biobank were employed to derive a weighted Lp(a) PRS for Lp(a) levels. The PRS comprised 43 SNVs that had been previously demonstrated to be significantly and conditionally linked with Lp(a) levels in external datasets (21). A previous study has demonstrated that this Lp(a) PRS had a comparable ability to predict cardiovascular events as plasma Lp(a) levels (20). Details of the included 43 SNVs are shown in supplemental Table S1. We calculated a weighted PRS using these SNVs, with each SNV weighted by its effect on circulating Lp(a) levels. Specifically, the Lp(a) PRS was calculated using the weighted formula as follows: PRS = (β_1_ × SNV_1_ + β_2_ × SNV_2_ + … + β_43_ × SNV_43_) × (43/sum of the β coefficients). The PRS for Lp(a) ranged from −140.76 to 388.40, with higher scores indicating elevated levels of circulating Lp(a).

### Ascertainment of HF

The identification of prevalent and incident cases of HF was accomplished by merging self-reported data and hospital inpatient records. The UK Biobank dataset was linked with Health Episode Statistics in England and Wales and the Scottish Morbidity Records in Scotland to determine the timing and diagnosis of hospital admissions. Diagnostic criteria for prevalent and incident cases were established using the International Classification of Diseases (ICD) codes ICD-9: 4280, 4281, and 4289; and ICD-10: I11.0, I13.0, I13.2, I50.0, I50.1, and I50.9, as presented in supplemental Table S2. The hospital inpatient records were available until November 30, 2020, for the present analysis, while the most recent updates to mortality data for England and Wales and Scotland were on December 18, 2020, and December 10, 2020, respectively. These dates were considered to be the end of the follow-up period where appropriate. Person time was calculated from the baseline assessment date until the earliest occurrence of an event diagnosis, death, loss to follow-up, or the end of the follow-up period.

### Assessment of classic risk factors

In the multivariable-adjusted models, we used the classic risk factors as covariates for HF according to the Atherosclerosis Risk in Communities (ARIC) study-sans-BNP score (i.e., without BNP/NT-proBNP) (22). These classic risk factors included age, sex, ethnicity (coded as White, Black or Black British, Asian or Asian British, Chinese, mixed, and other ethnic groups), body mass index (BMI), smoking status (never, past, or current), systolic blood pressure (SBP), antihypertensive medication use, diabetes, and heart rate. The ARIC sans-BNP score provides validated classic HF risk factors and is likely the most widely used model in clinical practice (23). Notably, CVD history was another component risk factor in ARIC sans-BNP score; however, to better generalize our findings to the general population and better facilitate comparison with findings from previous studies on this topic (6, 8, 20, 24), we excluded those with prevalent CVD at baseline (described below).

The height of the study participants was ascertained utilizing a nonstretchable measuring tape, whereas weight measurements were obtained using the Tanita BC-418 MA body analyzer (25). BMI was calculated as the quotient of weight in kilograms and height in meters squared (kg/m^2^). Trained nurses collected baseline blood pressure measurements by employing a digital blood pressure monitor (Omron HEM-7015IT; OMRON Healthcare Europe B.V) with an appropriately sized cuff, following a minimum of 5 min of seated rest for participants. The arithmetic means of SBP, DBP, and heart rate values were calculated from two automated or two manual measurements administered by the trained nurses (26). Hypertension was defined as having an SBP >140 mmHg, DBP exceeding 90 mmHg, or being on antihypertensive medication. Diabetes status was ascertained by self-reported history of diabetes, self-reported use of diabetes medication, or an HbA1c level equal to or greater than 48 mmol/mol (6.5%). All biochemical assays, including measurements of serum total cholesterol (Beckman Coulter AU5800) and HbA1c levels (VARIANT II Turbo; Bio-Rad, Hercules, California), were conducted in a dedicated central laboratory during the period spanning from November 05, 2014, to October 03, 2017. The UK Biobank blood collection procedures have undergone validation (27), and a comprehensive description of the measurements is available online (http://www.ukbiobank.ac.uk).

### Statistical analysis

We excluded participants who had self-reported or clinically diagnosed HF (n = 2,405) or CVD (myocardial infarction [MI], angina, or stroke) (n = 32,860) at baseline. We further excluded participants who missed data on Lp(a) (n = 116,610), FHx of CVD (n = 47,839), or HF classic risk factors (n = 3,228). After these exclusions, 299,158 participants remained for our analyses. For the analyses involving PRS, we further excluded non-White participants (n = 14,949) and those without genetic information (n = 3,352), leaving an analytic sample of 280,857 participants.

We used Cox proportional hazards models to estimate hazard ratios (HRs) and 95% confidence intervals (CIs) of HF for Lp(a) categories and different types of FHx of CVD. Given the well-characterized racial differences in Lp(a) concentrations (28), circulating Lp(a) concentration was categorized into ethnicity-specific quintiles. To better translate our results into clinical practice, we also grouped the sample using the circulating Lp(a) cut-off of 105 nmol/L (50 mg/dl) (29). We used the abovementioned classic risk factors to construct the model, and Lp(a) and FHx were mutually adjusted for each other. The statistical significance of the Lp(a)-FHx interaction on HF risk was tested by adding a multiplicative term to the multivariable models. In addition, FHx of CVD and circulating ethnicity-specific Lp(a) quintile or circulating Lp(a) cut-off category were combined to examine the joined effects of FHx and circulating Lp(a) on the risk of HF. For this joint association analysis, we conducted subgroup analysis according to age (<60 or ≥ 60 years), sex, ethnicity (White/non-White), BMI (<30 or ≥30 kg/m^2^), ever smoke (no or yes), hypertension, diabetes, and total cholesterol level (<5.17 or ≥5.17 mmol/L). Sensitivity analysis was also performed by further adjusting for apolipoprotein B [(apo) B], as previous studies have reported that apoB may be the primary driver of atherosclerosis beyond other lipids (30, 31). To facilitate comparisons with previous studies and to explore whether other major heart diseases may mediate the link between Lp(a) and HF (6, 7), we excluded participants with prevalent or incident aortic valve stenosis or incident MI at any time before (or at the time of) HF diagnosis during follow-up. The abilities of Lp(a) and FHx in predicting HF risk were assessed by adding either or both in the multivariable Cox proportional hazard models including classic risk factors. Harrell’s C-statistic was used to assess the discrimination (32), while classification improvement was assessed by the continuous net reclassification improvement (NRI) index for 10-years follow-up (33, 34). Where appropriate, we also repeated the abovementioned analyses by using Lp(a) PRS as the exposure of interest, while adjusting for the predefined covariates plus the first 10 principal components of ancestry.

STATA 14.0 (StataCorp LP) was used for most of the analyses, while the C-index and NRI were estimated using R statistical software, specifically the "CsChange" and "nricens" packages. A two-sided *P* value <0.05 was considered statistically significant.

## Results

### Baseline participant characteristics

For most HF classic risk factors, no clear trends were observed across circulating ethnicity-specific quintiles of Lp(a) (Table 1). However, the percentage of positive FHx and Lp(a) PRS increased with increasing circulating Lp(a) concentrations. Similarly, those with higher Lp(a) PRS tended to have positive FHx and higher circulating Lp(a) concentrations (supplemental Table S3). On the other hand, as compared with participants who reported no CVD history among their family members, those who reported a positive history of CVD in all family members tended to be older, were more likely to be women, non-smokers, users of antihypertensive medications and statins, and have diabetes, and they also have higher SBP levels and Lp(a) PRS (supplemental Table S4). For White and Asian/Asian British, the concentrations of Lp(a) were higher among those who reported a positive history of CVD in their parents or siblings than those who did not (supplemental Table S4). Participant characteristics by parental history of CVD alone (supplemental Table S5) and sibling history of CVD alone (supplemental Table S6) are presented as supplementary data.

**Table 1:** Baseline participant characteristics by ethnicity-specific quintiles of circulating Lp(a) concentration

	Circulating Lp(a) Q1 (n = 60,057)	Circulating Lp(a) Q2 (n = 59,784)	Circulating Lp(a) Q3 (n = 59,731)	Circulating Lp(a) Q4 (n = 59,782)	Circulating Lp(a) Q5 (n = 59,804)
Lp(a), nmol/L	5.70 (3.80–33.6)	11.0 (8.0–57.6)	20.5 (12.5–79.3)	47.6 (22.3–117.2)	130.3 (50.8–189.0)
Lp(a) PRS	0.43 (−140.8 to 309.6)	7.3 (−130.7 to 343.2)	14.6 (−119.4 to 376.1)	27.7 (−127.1 to 332.0)	113.5 (−35.6 to 388.4)
Age, y	56.0 (49.0–62.0)	57.0 (49.0–62.0)	57.0 (50.0–63.0)	58.0 (50.0–63.0)	57.0 (49.0–62.0)
Men, %	45.4	44.8	41.4	39.0	42.8
Ethnicity					
White, %	94.9	94.9	94.9	94.9	94.9
Black or Black British, %	1.4	1.4	1.4	1.4	1.4
Asian or Asian British, %	1.9	1.9	1.9	1.9	1.9
Chinese, %	0.3	0.3	0.3	0.3	0.3
Mixed, %	0.6	0.6	0.6	0.6	0.6
Other, %	0.9	0.9	0.9	0.9	0.9
BMI, kg/m^2^	26.5 (23.9–29.6)	26.4 (23.9–29.5)	26.4 (23.9–29.4)	26.5 (24.0–29.5)	26.5 (23.9–29.5)
Current smoker, %	10.2	10.0	9.7	9.8	10.0
SBP, mmHg	137.0 (125.0–151.0)	137.0 (125.0–151.0)	137.0 (125.0–151.0)	138.0 (125.0–151.0)	137.0 (125.0–151.0)
Antihypertensive medication use, %	16.9	16.4	16.4	16.8	16.2
Heart rate, bpm	69.0 (62.0–77.0)	69.0 (62.0–76.0)	68.0 (62.0–76.0)	69.0 (62.0–76.0)	69.0 (62.0–76.0)
Diabetes, %	5.8	4.7	4.2	4.2	4.5
Parents had CVD, %	38.3	39.1	40.0	41.3	43.0
Siblings had CVD, %	7.0	7.1	7.7	8.0	8.1
Parents or siblings had CVD, %	40.9	41.6	42.7	44.2	45.9

BMI, body mass index; CVD, cardiovascular disease; Lp(a), lipoprotein(a); SBP, systolic blood pressure. Continuous variables are described as median (interquartile range) and categorical variables are described as percentages. Circulating Lp(a) concentrations and Lp(a) PRS are described as median (range). Lp(a) PRS was estimated among the white only.

### Association of Lp(a) and FHx with HF

Over a median of 11.8 years of follow-up (interquartile range: 11.1–12.5 years), there were 5,502 HF cases. Higher concentrations of circulating Lp(a) and all types of positive FHx were associated with higher risks of HF. The adjusted HR (95% CI) of HF was 1.15 (1.06, 1.25) comparing the highest with the lowest circulating ethnicity-specific quintile of Lp (a) (*P* for trend <0.001). We also found that compared with the lowest quintile of Lp(a) PRS, those with the highest quintile of Lp(a) PRS had elevated risk of HF, with adjusted HR (95% CI) of 1.16 (1.06, 1.26) (*P* for trend =0.002). Using the clinical cut-off and comparing Lp(a) ≥105 nmol/L with Lp(a) <105 nmol/L, the adjusted HR (95% CI) was 1.13 (1.06, 1.20). As compared with participants without FHx of CVD, participants who reported a positive history of CVD among all family members had 19% higher risk of HF, and the increases in the risk were 13% for those with a parental history of CVD and 22% for those with a sibling history of CVD (Table 2). Similar results were also noted regarding FHx and HF risk among the White who had available genetic data.

**Table 2:** Hazard ratios for HF according to the quintiles of Lp(a), clinically relevant cutoffs, or family history of CVD

	Circulating Lp(a) & FHx		Lp(a) PRS & FHx	
	Events/Person-y	Adjusted HR (95% CI)	Events/Person-y	Adjusted HR (95% CI)
By quintiles				
Q1	1,083/696,244	Ref.	965/652,411	Ref.
Q2	1,033/693,884	0.98 (0.90, 1.07)	1,028/652,335	1.05 (0.96, 1.14)
Q3	1,080/692,513	1.03 (0.95, 1.12)	1,055/651,704	1.06 (0.97, 1.16)
Q4	1,120/691,895	1.06 (0.97, 1.15)	1,049/651,453	1.07 (0.98, 1.16)
Q5	1,186/693,346	1.15 (1.06, 1.25)	1,112/652,351	1.16 (1.06, 1.26)
*P* for trend	−	<0.001	−	0.002
By quintiles				
Q1-Q4	4,316/2,774,538	Ref.	4,097/2,607,903	Ref.
Q5	1,186/693,346	1.13 (1.06, 1.20)	1,112/652,351	1.11 (1.04, 1.19)
Parents or siblings had CVD				
No	2,732/1,974,579	Ref.	2,551/1,839,011	Ref.
Yes	2,770/1,493,305	1.19 (1.13, 1.26)	2,658/1,421,243	1.20 (1.14, 1.27)
Parents had CVD				
No	3,002/2,068,690	Ref.	2,798/1,926,197	Ref.
Yes	2,500/1,399,194	1.13 (1.07, 1.20)	2,411/1,334,057	1.15 (1.09, 1.21)
Siblings had CVD				
No	4,789/3,207,841	Ref.	4,537/3,016,883	Ref.
Yes	713/260,043	1.22 (1.12, 1.32)	672/243,371	1.21 (1.12, 1.32)
By Lp(a) cut-off				
Lp(a) <105 nmol/L	4,595/2,943,932	Ref.	−	−
Lp(a) ≥105 nmol/L	907/523,952	1.13 (1.06, 1.22)	−	−

CI, confidence interval; HR, hazard ratios; Lp(a), Lipoprotein(a); PRS, polygenetic risk score. HRs were adjusted for ARIC sans-BNP score, including age, sex, ethnicity, smoking status, BMI, SBP, antihypertensive medication use, heart rate, and diabetes; and Lp(a) and FHx were mutually adjusted for. For the analyses regarding genetic data, the analyses were conducted among the White, and the first 10 principal components of ancestry were further adjusted for.

**Fig. 1:**
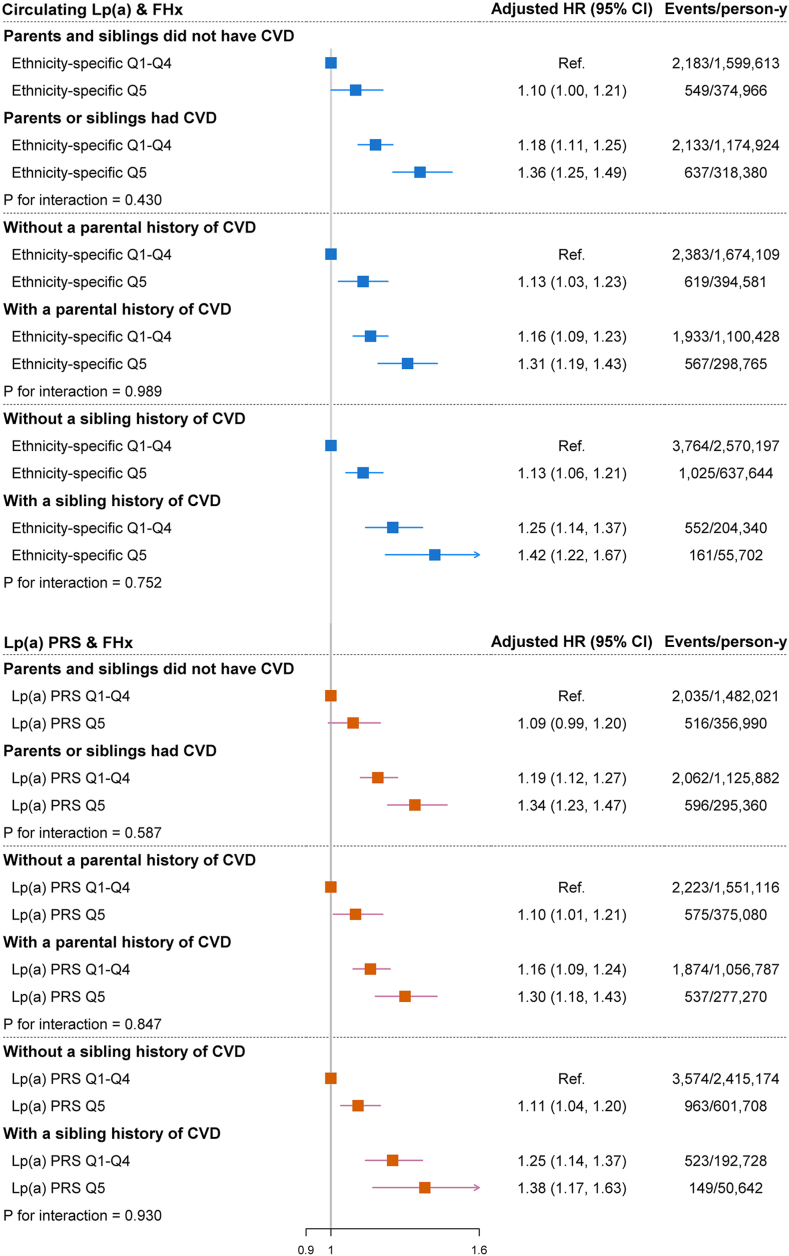
Hazard ratios for the joint associations of quintiles for Lp(a) and different types of FHx of CVD with incident HF. HRs were adjusted for ARIC sans-BNP score, including age, sex, ethnicity, smoking status, BMI, SBP, antihypertensive medication use, heart rate, and diabetes. For Lp(a) PRS, the first 10 principal components of ancestry were further adjusted for.

There were no significant interactions between elevated circulating Lp(a)/Lp(a) PRS and any types of FHx on risk of HF (all *P* for interactions >0.16) (supplemental Tables S7–S9). In the joint analysis of Lp(a) and FHx of CVD, those with both elevated Lp(a) (top quintile of circulating Lp[a] or Lp[a] PRS) and positive FHx had the highest risk of HF. As compared with non-elevated circulating Lp(a) (the first to the fourth quintile) and negative FHx of CVD, the adjusted HRs (CIs) were 1.36 (1.25, 1.49), 1.31 (1.19, 1.43), and 1.42 (1.22, 1.67), respectively, for the combination of elevated Lp(a) and positive history of CVD in all family members, in parents, or in siblings; the corresponding values for Lp(a) PRS were 1.34 (1.23, 1.47), 1.30 (1.18, 1.43), and 1.38 (1.17, 1.63), respectively (Fig. 1). These joint associations were also seen when the Lp(a) clinical cut-off of 105 nmol/L were used instead of circulating Lp(a) quintiles (supplemental Fig. S1).

The joint association of circulating Lp(a)/Lp(a) PRS and all FHx of CVD with incident HF were observed across various population subgroups defined by age, sex, ethnicity, BMI, smoking status, hypertension, and diabetes (supplemental Tables S10 and S11). The combined association did not materially change after further adjustment for apolipoprotein B [apo(B)] concentrations. In sensitivity analyses aimed at evaluating the potential mediating role of aortic valve stenosis and MI, we found that the risk estimates for HF among those with elevated Lp(a) and positive FHx remained almost unchanged after excluding those with prevalent or incident aortic valve stenosis. However, when excluding participants with incident MI, the risk estimates for HF were attenuated; notably, the magnitude of attenuation remained almost the same as excluding incident MI when prevalent and incident aortic valve stenosis and incident MI were simultaneously excluded (Tables 3 and 4).

**Table 3:** Sensitivity analysis for the joint associations of circulating Lp(a) and FHx of CVD with risk of incident HF

Parents or Siblings had CVD				
	No		Yes	
	Circulating Ethnicity-specific Q1-Q4	Circulating Ethnicity-specific Q5	Circulating Ethnicity-specific Q1-Q4	Circulating Ethnicity-specific Q5
Further adjustment for apo(B)				
Events/person-y	2,183/1,599,613	549/374,966	2,133/1,174,924	637/318,380
HR (95% CI)	Ref.	1.10 (1.01, 1.21)	1.18 (1.11, 1.25)	1.37 (1.26, 1.50)
Exclusion of participants with prevalent or incident aortic valve stenosis				
Events/person-y	2,087/1,592,215	526/372,441	2,035/1,166,999	604/315,397
HR (95% CI)	Ref.	1.10 (1.00, 1.21)	1.18 (1.11, 1.26)	1.37 (1.25, 1.50)
Exclusion of participants with incident MI				
Events/person-y	2,007/1,576,931	493/368,087	1,926/1,149,994	541/309,345
HR (95% CI)	Ref.	1.09 (0.98, 1.19)	1.16 (1.09, 1.24)	1.28 (1.17, 1.41)
Exclusion of participants with prevalent, incident aortic valve stenosis or incident MI				
Events/person-y	1,923/1,570,053	474/365,773	1,842/1,142,931	515/306,862
HR (95% CI)	Ref.	1.08 (0.98, 1.20)	1.17 (1.09, 1.24)	1.28 (1.17, 1.42)

apo(B), apolipoprotein B; FHx, family history; HF, heart failure; MI, myocardial infarction. Adjustments were made for ARIC sans-BNP score, including age, sex, ethnicity, smoking status, BMI, SBP, antihypertensive medication use, heart rate, and diabetes, unless otherwise specified.

**Table 4:** Sensitivity analysis for the joint associations of Lp(a) PRS and FHx of CVD with risk of incident HF among the White

Parents or Siblings had CVD				
	No		Yes	
	Lp(a) PRS Q1-Q4	Lp(a) PRS Q5	Lp(a) PRS Q1-Q4	Lp(a) PRS Q5
Further adjustment for apo(B)				
Events/person-y	2,035/1,482,021	516/356,990	2,062/1,125,882	596/295,360
HR (95% CI)	Ref.	1.09 (0.99, 1.20)	1.19 (1.12, 1.27)	1.34 (1.23, 1.47)
Exclusion of participants with prevalent or incident aortic valve stenosis				
Events/person-y	1,946/1,474,960	493/354,513	1,974/1,118,144	559/292,572
HR (95% CI)	Ref.	1.09 (0.99, 1.20)	1.20 (1.12, 1.27)	1.34 (1.21, 1.47)
Exclusion of participants with incident MI				
Events/person-y	1862/1,460,869	474/350,699	1854/1,102,078	517/287,062
HR (95% CI)	Ref.	1.10 (0.99, 1.22)	1.18 (1.10, 1.26)	1.30 (1.18, 1.43)
Exclusion of participants with prevalent, incident aortic valve stenosis or incident MI				
Events/person-y	1785/1,454,326	454/348,407	1778/1,095,154	487/284,767
HR (95% CI)	Ref.	1.10 (0.99, 1.22)	1.18 (1.10, 1.26)	1.30 (1.16, 1.42)

apo(B), apolipoprotein B; FHx, family history; HF, heart failure; MI, myocardial infarction; PRS, polygenetic risk score. Adjustments were made for ARIC sans-BNP score, including age, sex, ethnicity, smoking status, BMI, SBP, antihypertensive medication use, heart rate, diabetes and the first 10 principal components of ancestry, unless otherwise specified.

### Prediction of HF

**Fig. 2:**
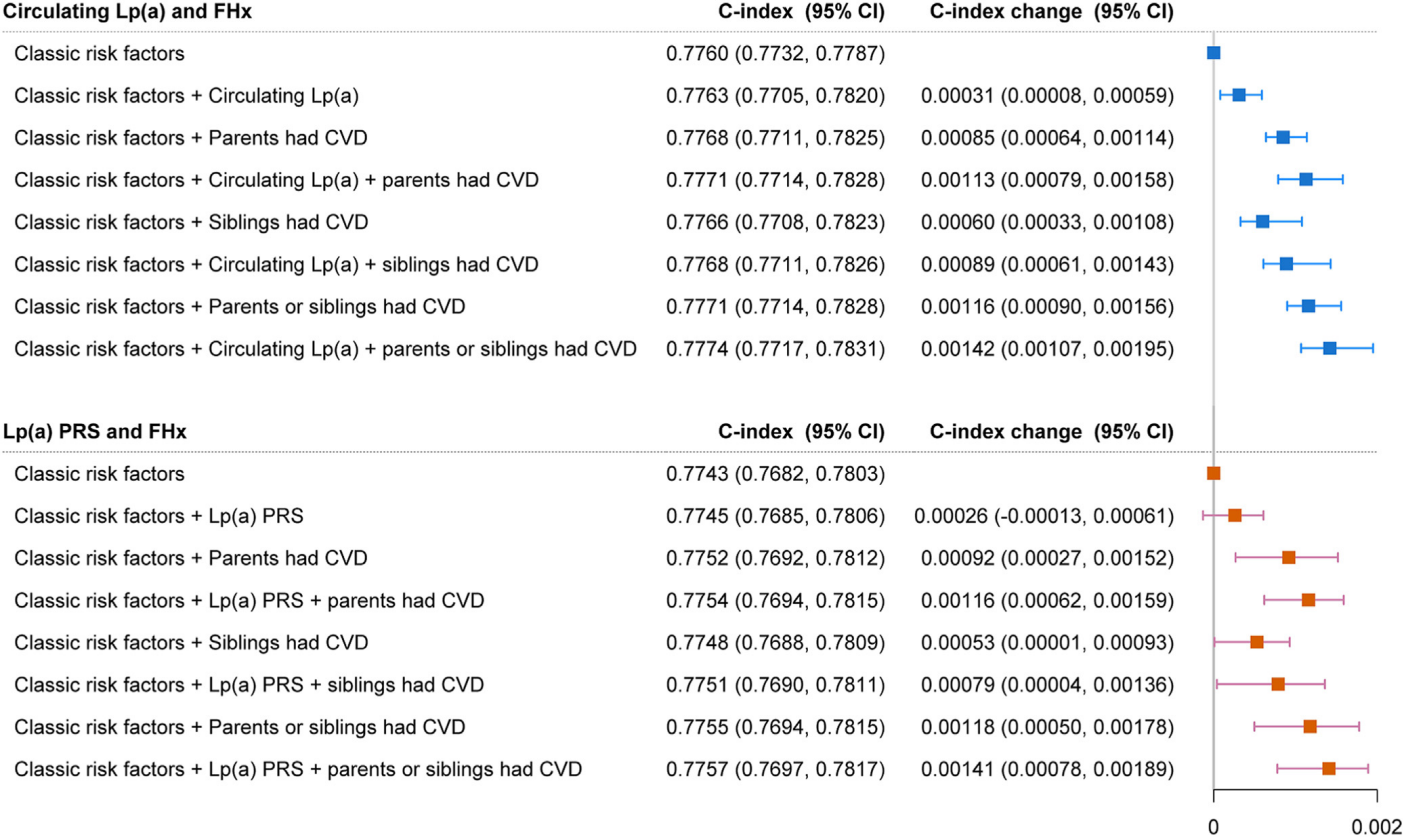
Change in C-index for HF with the addition of Lp(a) and different types of FHx of CVD. Classic risk factors refer to ARIC sans-BNP score, including age, sex, ethnicity, smoking status, BMI, SBP, antihypertensive medication use, heart rate, and diabetes.

ARIC-sans-BNP risk factors yielded a C-index of 0.7760 (95% CI 0.7732, 0.7787) and 0.7743 (95% CI 0.7682, 0.7809) for analyses regarding circulating Lp(a) and Lp(a) PRS, respectively. Adding the circulating Lp(a) or all types of FHx resulted in an improvement of C-index, with a change of 0.00031, 0.00085, 0.00060, and 0.00116 for circulating Lp(a), parental history, sibling history, and all FHx of CVD, respectively. Although Lp(a) PRS did not result in an improvement of the C-index, all the FHx provide an improvement of the C-index. When circulating Lp(a)/Lp(a) PRS and all types of FHx were added to the model simultaneously, the combination of both factors yield best improvements than either one alone (Fig. 2). Similar improvements of risk classification were observed (supplemental Table S12). Specifically, the addition of both circulating Lp(a)/Lp(a) PRS and FHx resulted in better improvements of reclassification than either marker alone.

## Discussion

In the present study of UK population without baseline HF and other major CVD, we observed that both elevated Lp(a), in terms of circulating concentration or Lp(a) PRS, and a positive FHx of CVD (parents, siblings, or both) were independently associated with a higher risk of HF. Furthermore, those with both elevated Lp(a) and positive FHx had the highest risk of HF, compared with those having neither risk factors. The risk estimates for HF were attenuated after excluding participants who had incident MI during follow-up. The addition of Lp(a) and FHx history of CVD in parents or siblings to a classic risk factor model appeared to improve HF risk discrimination and reclassification better than adding one marker alone.

The biological mechanisms that link Lp(a) to HF risk are not fully understood. Evidence from in vitro, animal, and large genetic association studies has shown that extreme levels of Lp(a) may promote atherosclerotic stenosis and thrombosis (35, 36, 37, 38), which may ultimately lead to coronary heart disease (CHD) and HF. For example, Lp(a) can cross the endothelial barrier and retain in the injured arterial intima, and it may promote foam cell formation and smooth muscle cell proliferation as well as plaque inflammation and instability, and thereby atherosclerosis (38, 39, 40, 41).

A few prospective cohort studies have investigated the association between Lp(a) and incident HF, but the results have been mixed (6, 7, 8). Kamstrup *et al.* combined two Danish Copenhagen-based cohorts and found that elevated circulating Lp(a) and related genetic information (e.g., LPA KIV-2 genotype) were both linked to a higher risk of HF, after excluding those with prevalent MI or aortic valve stenosis at baseline (6). In the ARIC study, although Agarwala *et al.* found an increased risk of HF associated with elevated Lp(a), the results became non-significant after excluding those with prevalent or incident MI (8). Our results confirm a positive association between Lp(a) and HF in a large, multiethnic population without prevalent major CVD, as evidenced by both circulating Lp(a) concentrations and Lp(a) PRS. Notably, the risk estimates for HF were attenuated after excluding participants with incident MI but not those with prevalent or incident aortic valve stenosis. These results suggest that the excess risk of HF associated with Lp(a) and FHx may be partly mediated by incident MI during follow-up.

It has been well-documented that Lp(a) levels vary across race/ethnicity, and the association between Lp(a) and HF risk may differ across different race/ethnicity groups. For instance, in the Multi-Ethnic Study of Atherosclerosis (MESA) study, which included Caucasian, Black, Hispanic, and Chinese populations, Steffen *et al.* found that the Lp(a)–HF association was modified by race/ethnicity and that the positive association was limited to Caucasian participants (7). In contrast, some previous studies have reported that elevated Lp(a) was linked to HF risk among Asians, including Chinese and Japanese (42, 43). In our study, we did not find evidence of race/ethnicity interaction. However, as the majority of the UK Biobank population was White, it is possible that the significant associations we observed were driven by findings in White individuals. Further research is necessary to investigate the potential mechanisms that may underlie the race/ethnicity-based disparities in Lp(a)-associated risk.

It has been widely demonstrated that genetic factors contribute significantly to the variation in circulating Lp(a) levels, and FHx captures both the cumulative environmental exposures over a lifetime and the polygenic predisposition to CVD. In fact, multiple studies have firmly established Lp(a) and FHx of CVD as well-established risk factors for the development of CVD or HF in the general population (9, 11, 12, 14, 44). As such, both American and European medical societies advocate for the assessment of Lp(a) concentrations among individuals with an FHx of CVD (15, 16). However, it has been less clear whether the coexistence of elevated Lp(a) and positive FHx represents a particularly high-risk phenotype. Durrington *et al.* conducted a case-control study involving 119 White men, wherein they observed that apo(a) concentration accounted for a significant portion of the familial predisposition to CHD. Based on their findings, the authors concluded that apo(a) and parental history could be used interchangeably as risk factors for CHD (45). Similarly, Mehta *et al.* reported in their investigation of 12,149 middle-aged and older adults from the ARIC study that elevated plasma Lp(a) and positive FHx of CVD had independent and additive joint associations with CVD risk (17). In that study, those with either elevated Lp(a) (highest quintile) or a parental history of CVD had a higher risk of ASCVD and CHD, and those with both had the highest risk (ASCVD: HR =1.43; CHD: HR =1.68), as compared with participants with lower Lp(a) (quintile 1–4) and no parental history of CVD. However, this study did not focus on HF and only considered the parental history of CVD.

Lp(a) and FHx of CVD are two genetic-related factors, however, FHx of CVD may have distinct genetic bases in comparison to Lp(a), as the environmental and lifestyle factors may also play important roles. Indeed, traditional risk factors always have a tendency of familial aggregation and parental history of CVD or sibling history of CVD are linked to unhealthy lifestyles and adverse lipid profiles (46, 47, 48). Given that there are no approved medications to specifically lower Lp(a) so far (49, 50), current strategies for the management of individuals with high Lp(a) may primarily focus on managing modifiable risk factors among high-risk populations. Therefore, for patients with elevated Lp(a), clinicians should focus on proven lifestyles to modify traditional risk factors and reduce HF risk, especially among those with a positive FHx of CVD.

It is also noteworthy that, at present, there is no HF risk score that incorporates both Lp(a) and FHx of CVD. To address this gap, we investigated the predictive value of Lp(a) and FHx of CVD using the widely acknowledged ARIC sans-BNP model, an HF risk prediction tool. Although our study revealed a modest improvement in prediction ability with the addition of Lp(a) and FHx of CVD (e.g., the improvement in C-index), our findings offer novel insights into the risk stratification and prediction of HF in the general population.

### Strengths and limitations

The present study was one of the largest studies examining the relationship between Lp(a) and incident HF and, to our knowledge, the first study to investigate the combined effect of circulating Lp(a) and Lp(a) PRS and FHx of CVD on HF risk. The linkage with hospital registries and death records assured complete information on the endpoints and minimized the attrition rate. Nevertheless, several limitations must be taken into account when interpreting the findings of this study. First, the UK Biobank cohort was not a representative sample of the United Kingdom population, potentially constraining the generalizability of our results. Moreover, given the predominance of White ethnicity within the cohort, the validation of our findings in other ethnic groups is needed. Second, although premature FHx of CVD (e.g., age of onset of CVD <55 years) may provide more information on environmental and polygenic exposures (17), the UK biobank did not collect information about the age of onset of CVD for participants’ relatives; thus, we could not examine the association of early-onset of CVD in parents or siblings with HF risk. Third, FHx was self-reported, which might affect the accuracy of the exposure and lead to misclassification bias. Fourth, the UK Biobank did not capture subtypes of HF, specifically HF with reduced ejection fraction (HFrEF) and HF with preserved ejection fraction (HFpEF), which may have different underlying pathophysiology (51, 52). Additional studies are needed to investigate whether the associations of Lp(a) and FHx of CVD with HF differ by HF subtypes. Finally, it is important to note that our sensitivity analyses have revealed that coronary artery disease resulting from elevated levels of Lp(a) may directly lead to ischemia and HF, independent of the occurrence of MI. However, the precise underlying mechanisms responsible for the Lp(a)-HF link remained elusive. Further investigations are warranted to discern the specific pathways implicated in the development of HF in the context of elevated Lp(a).

## Conclusion

In a prospective cohort of UK population without baseline major CVD, circulating Lp(a), Lp(a) PRS, and FHx of CVD were found to be independent risk factors for incident HF. The highest risk of HF was observed among those with both elevated Lp(a) and positive FHx, and this association may be partly mediated by MI.

## Data Availability

Data is available from the UK Biobank Institutional Data Access / Ethics Committee (contact via http://www.ukbiobank.ac.uk/ or contact by email at ku.ca.knaboibku@ssecca) for researchers who meet the criteria for access to confidential data.

## Supplemental data

This article contains supplemental data (20, 21).

## Conflict of interest

The authors declare that they have no known competing financial interests or personal relationships that could have appeared to influence the work reported in this paper.

## References

[1] Heidenreich P.A., Albert N.M., Allen L.A., Bluemke D.A., Butler J., Fonarow G.C. (2013). Forecasting the impact of heart failure in the united states: a policy statement from the american heart association. Circ. Heart Fail..

[2] Roger V.L. (2013). Epidemiology of heart failure. Circ. Res..

[3] Cowie M.R., Wood D.A., Coats A.J., Thompson S.G., Suresh V., Poole-Wilson P.A. (2000). Survival of patients with a new diagnosis of heart failure: a population based study. Heart.

[4] Mahley R.W., Innerarity T.L., Rall S.J., Weisgraber K.H. (1984). Plasma lipoproteins: apolipoprotein structure and function. J. Lipid Res..

[5] MBewu A.D., Durrington P.N. (1990). Lipoprotein (a): structure, properties and possible involvement in thrombogenesis and atherogenesis. Atherosclerosis.

[6] Kamstrup P.R., Nordestgaard B.G. (2016). Elevated Lipoprotein(a) levels, LPA risk genotypes, and increased risk of heart failure in the general population. JACC Heart Fail..

[7] Steffen B.T., Duprez D., Bertoni A.G., Guan W., Tsai M.Y. (2018). Lp(a) [Lipoprotein(a)]-related risk of heart failure is evident in whites but not in other racial/ethnic groups. Arterioscler. Thromb. Vasc. Biol..

[8] Agarwala A., Pokharel Y., Saeed A., Sun W., Virani S.S., Nambi V. (2017). The association of Lipoprotein(a) with incident heart failure hospitalization: atherosclerosis risk in communities study. Atherosclerosis.

[9] Lindgren M.P., PirouziFard M., Smith J.G., Sundquist J., Sundquist K., Zöller B. (2018). A swedish nationwide adoption study of the heritability of heart failure. JAMA Cardiol..

[10] van Oort S., Beulens J., van Ballegooijen A.J., Handoko M.L., Larsson S.C. (2020). Modifiable lifestyle factors and heart failure: a Mendelian randomization study. Am. Heart J..

[11] Lloyd-Jones D.M., Nam B.H., D'Agostino R.S., Levy D., Murabito J.M., Wang T.J. (2004). Parental cardiovascular disease as a risk factor for cardiovascular disease in middle-aged adults: a prospective study of parents and offspring. JAMA.

[12] Lee D.S., Pencina M.J., Benjamin E.J., Wang T.J., Levy D., O'Donnell C.J. (2006). Association of parental heart failure with risk of heart failure in offspring. N. Engl. J. Med..

[13] Lindgren M.P., Smith J.G., Li X., Sundquist J., Sundquist K., Zöller B. (2018). Familial mortality risks in patients with heart failure-a swedish sibling study. J. Am. Heart Assoc..

[14] Lindgren M.P., Smith J.G., Li X., Sundquist J., Sundquist K., Zöller B. (2016). Sibling risk of hospitalization for heart failure - A nationwide study. Int. J. Cardiol..

[15] Mach F., Baigent C., Catapano A.L., Koskinas K.C., Casula M., Badimon L. (2020). 2019 ESC/EAS Guidelines for the management of dyslipidaemias: lipid modification to reduce cardiovascular risk. Eur. Heart J..

[16] Jacobson T.A., Ito K.C., Maki C.E., Orringer C.E., Bays H.E., Jones P.H. (2015). National lipid association recommendations for patient-centered management of dyslipidemia: part 2. J. Clin. Lipidol..

[17] Mehta A., Virani S.S., Ayers C.R., Sun W., Hoogeveen R.C., Rohatgi A. (2020). Lipoprotein(a) and family history predict cardiovascular disease risk. J. Am. Coll. Cardiol..

[18] (2018). UK Biobank data on 500,000 people paves way to precision medicine. Nature.

[19] Bycroft C., Freeman C., Petkova D., Band G., Elliott L.T., Sharp K. (2018). The UK Biobank resource with deep phenotyping and genomic data. Nature.

[20] Trinder M., Uddin M.M., Finneran P., Aragam K.G., Natarajan P. (2020). Clinical utility of Lipoprotein(a) and LPA genetic risk score in risk prediction of incident atherosclerotic cardiovascular disease. JAMA Cardiol..

[21] Burgess S., Ference B.A., Staley J.R., Freitag D.F., Mason A.M., Nielsen S.F. (2018). Association of LPA variants with risk of coronary disease and the implications for Lipoprotein(a)-lowering therapies: a mendelian randomization analysis. JAMA Cardiol..

[22] Agarwal S.K., Chambless L.E., Ballantyne C.M., Astor B., Bertoni A.G., Chang P.P. (2012). Prediction of incident heart failure in general practice. Circ. Heart Fail..

[23] Nowak C., Arnlov J. (2020). Kidney disease biomarkers improve heart failure risk prediction in the general population. Circ. Heart Fail..

[24] Virani S.S., Brautbar A., Davis B.C., Nambi V., Hoogeveen R.C., Sharrett A.R. (2012). Associations between Lipoprotein(a) levels and cardiovascular outcomes in black and white subjects. Circulation.

[25] Li F.R., Yang H.L., Zhou R., Zheng J.Z., Chen G.C., Zou M.C. (2021). Diabetes duration and glycaemic control as predictors of cardiovascular disease and mortality. Diabetes Obes. Metab..

[26] Li F.R., He Y., Yang H.L., Liu H.M., Zhou R., Chen G.C. (2021). Isolated systolic and diastolic hypertension by the 2017 American College of Cardiology/American Heart Association guidelines and risk of cardiovascular disease: a large prospective cohort study. J. Hypertens..

[27] Elliott P., Peakman T.C. (2008). The UK Biobank sample handling and storage protocol for the collection, processing and archiving of human blood and urine. Int. J. Epidemiol..

[28] Paré G., Çaku A., McQueen M., Anand S.S., Enas E., Clarke R. (2019). Lipoprotein(a) levels and the risk of myocardial infarction among 7 ethnic groups. Circulation.

[29] Madsen C.M., Kamstrup P.R., Langsted A., Varbo A., Nordestgaard B.G. (2020). Lipoprotein(a)-lowering by 50 mg/dL (105 nmol/L) may be needed to reduce cardiovascular disease 20% in secondary prevention: a population-based study. Arterioscler. Thromb. Vasc. Biol..

[30] Marston N.A., Giugliano R.P., Melloni G.E.M., Park J.-G., Morrill V., Blazing M.A. (2021). Association of apolipoprotein B–containing lipoproteins and risk of myocardial infarction in individuals with and without atherosclerosis. JAMA Cardiol..

[31] Johannesen C.D.L., Mortensen M.B., Langsted A., Nordestgaard B.G. (2021). Apolipoprotein B and Non-HDL cholesterol better reflect residual risk than LDL cholesterol in statin-treated patients. J. Am. Coll. Cardiol..

[32] Harrell F.J., Lee K.L., Mark D.B. (1996). Multivariable prognostic models: issues in developing models, evaluating assumptions and adequacy, and measuring and reducing errors. Stat. Med..

[33] Pencina M.J., D'Agostino R.S., Steyerberg E.W. (2011). Extensions of net reclassification improvement calculations to measure usefulness of new biomarkers. Stat. Med..

[34] Kerr K.F., Wang Z., Janes H., McClelland R.L., Psaty B.M., Pepe M.S. (2014). Net reclassification indices for evaluating risk prediction instruments: a critical review. Epidemiology.

[35] Kamstrup P.R., Tybjaerg-Hansen A., Steffensen R., Nordestgaard B.G. (2009). Genetically elevated Lipoprotein(a) and increased risk of myocardial infarction. JAMA.

[36] Clarke R., Peden J.F., Hopewell J.C., Kyriakou T., Goel A., Heath S.C. (2009). Genetic variants associated with Lp(a) lipoprotein level and coronary disease. N. Engl. J. Med..

[37] Kamstrup P.R., Tybjærg-Hansen A., Nordestgaard B.G. (2012). Genetic evidence that Lipoprotein(a) associates with atherosclerotic stenosis rather than venous thrombosis. Arterioscler. Thromb. Vasc. Biol..

[38] Kamstrup P.R. (2010). Lipoprotein(a) and ischemic heart disease--a causal association? A review. Atherosclerosis.

[39] Nielsen L.B. (1999). Atherogenecity of Lipoprotein(a) and oxidized low density lipoprotein: insight from in vivo studies of arterial wall influx, degradation and efflux. Atherosclerosis.

[40] Boffa M.B., Marcovina S.M., Koschinsky M.L. (2004). Lipoprotein(a) as a risk factor for atherosclerosis and thrombosis: mechanistic insights from animal models. Clin. Biochem..

[41] Deb A., Caplice N.M. (2004). Lipoprotein(a): new insights into mechanisms of atherogenesis and thrombosis. Clin. Cardiol..

[42] Shitara J., Kasai T., Konishi H., Endo H., Wada H., Doi S. (2019). Impact of Lipoprotein (a) levels on long-term outcomes in patients with coronary artery disease and left ventricular systolic dysfunction. Circ. J..

[43] Yan J., Pan Y., Xiao J., Ma W., Li L., Zhong M. (2019). High level of Lipoprotein(a) as predictor for recurrent heart failure in patients with chronic heart failure: a cohort study. Arq Bras Cardiol..

[44] Ranthe M.F., Carstensen L., Oyen N., Tfelt-Hansen J., Christiansen M., McKenna W.J. (2012). family history of premature death and risk of early onset cardiovascular disease. J. Am. Coll. Cardiol..

[45] Durrington P.N., Ishola M., Hunt L., Arrol S., Bhatnagar D. (1988). Apolipoproteins (a), AI, and B and parental history in men with early onset ischaemic heart disease. Lancet.

[46] Allen J.K., Blumenthal R.S. (1998). Risk factors in the offspring of women with premature coronary heart disease. Am. Heart J..

[47] Becker D.M., Yook R.M., Moy T.F., Blumenthal R.S., Becker L.C. (1998). Markedly high prevalence of coronary risk factors in apparently healthy African-American and white siblings of persons with premature coronary heart disease. Am. J. Cardiol..

[48] De Backer G., De Henauw S., Sans S., Nicaud V., Masana L., Visvikis S. (1999). A comparison of lifestyle, genetic, bioclinical and biochemical variables of offspring with and without family histories of premature coronary heart disease: the experience of the European Atherosclerosis Research Studies. J. Cardiovasc. Risk.

[49] Bittner V.A., Szarek M., Aylward P.E., Bhatt D.L., Diaz R., Edelberg J.M. (2020). Effect of Alirocumab on Lipoprotein(a) and cardiovascular risk after acute coronary syndrome. J. Am. Coll. Cardiol..

[50] Tsimikas S., Moriarty P.M., Stroes E.S. (2021). Emerging RNA therapeutics to lower blood levels of Lp(a): JACC focus seminar 2/4. J. Am. Coll. Cardiol..

[51] Shah S.J., Borlaug B.A., Kitzman D.W., McCulloch A.D., Blaxall B.C., Agarwal R. (2020). Research priorities for heart failure with preserved ejection fraction: national heart, lung, and blood institute working group summary. Circulation.

[52] Simmonds S.J., Cuijpers I., Heymans S., Jones E. (2020). Cellular and molecular differences between HFpEF and HFrEF: a step ahead in an improved pathological understanding. Cells.

