# Supplementary material for: Lipoprotein(a), family history of cardiovascular disease, and incidence of heart failure
Source: J Lipid Res. 2023 Jun 3;64(7):100398. doi: 10.1016/j.jlr.2023.100398 (PMC10339055; doi:10.1016/j.jlr.2023.100398)
Supplement: Supplementary material [file mmc1.docx]

| **Supplementary Table S1**. List of 43 genetic variants used in the LPA polygenetic risk score. | | | | | |
| --- | --- | --- | --- | --- | --- |
| rsID | Chromosome: Position (GRCh37/hg19) | Effect allele | Other allele | Conditional association with Lp(a), nmol/l Beta^*^ | Conditional association with Lp(a), nmol/l SE^*^ |
| rs74617384 | 6:160997118 | T | A | 91.160 | 1.075 |
| rs140570886 | 6:161013013 | C | T | 172.430 | 1.720 |
| rs73596816 | 6:161017363 | A | G | 41.280 | 1.290 |
| rs182443492 | 6:160891897 | A | C | 79.120 | 2.150 |
| rs369686024 | 6:161032800 | A | G | 41.280 | 1.720 |
| rs56393506 | 6:161089307 | T | C | 26.660 | 0.860 |
| rs151135411 | 6:160831796 | A | G | 149.425 | 5.805 |
| rs145099029 | 6:161292838 | C | A | 38.270 | 3.870 |
| rs41267813 | 6:160998199 | A | G | -126.420 | 6.235 |
| rs6916433 | 6:160890350 | T | A | -10.105 | 0.645 |
| rs783147 | 6:161137990 | A | G | -4.300 | 0.645 |
| rs41266379 | 6:160953137 | C | T | 15.265 | 1.505 |
| rs143461353 | 6:160954800 | T | C | 28.165 | 2.150 |
| rs142126734 | 6:160942926 | A | G | 16.125 | 1.075 |
| rs139609547^#^ | 6:160899049 | A | - | 9.460 | 0.860 |
| rs1835346 | 6:161162290 | G | A | 11.180 | 1.505 |
| rs4252152 | 6:161159366 | G | T | 19.565 | 1.935 |
| rs79246098 | 6:161078894 | C | T | 13.330 | 1.935 |
| rs139145675 | 6:160966559 | A | G | -48.375 | 5.160 |
| rs41259144 | 6:161022107 | T | C | -20.640 | 1.720 |
| rs9456551 | 6:161012805 | C | T | 7.740 | 0.430 |
| rs41267809 | 6:160953642 | G | A | -14.190 | 1.290 |
| rs34371670 | 6:161257953 | T | C | -18.060 | 1.505 |
| rs41269876 | 6:161070653 | A | C | -17.630 | 1.290 |
| rs141834709 | 6:160909667 | A | T | 18.705 | 2.150 |
| rs4252170 | 6:161162406 | C | T | 6.880 | 0.860 |
| rs138491411 | 6:161251940 | G | A | 10.750 | 1.720 |
| rs183815886 | 6:160720804 | C | G | 31.820 | 3.870 |
| rs117446263 | 6:160847571 | A | G | -11.180 | 1.290 |
| rs200684404 | 6:160543317 | T | C | 145.555 | 19.780 |
| rs200144324 | 6:160493099 | T | C | 175.225 | 24.295 |
| rs77337569 | 6:161087652 | G | T | 11.180 | 1.720 |
| rs186418835 | 6:161214526 | A | G | -20.855 | 3.225 |
| rs117534432 | 6:161177443 | T | C | 7.095 | 1.075 |
| rs200376184 | 6:161011999 | C | G | 37.625 | 5.805 |
| rs11753588 | 6:161189071 | A | G | -5.160 | 0.645 |
| rs4709474 | 6:161285760 | G | A | 3.655 | 0.430 |
| rs191690882 | 6:161031132 | A | G | -28.380 | 4.085 |
| rs182349273 | 6:161255668 | G | A | 73.960 | 12.255 |
| rs75274517 | 6:161088956 | A | G | -13.975 | 2.150 |
| rs143365644 | 6:160825930 | T | A | 7.955 | 1.075 |
| rs139389770 | 6:161135746 | G | T | -11.180 | 1.935 |
| rs140606700 | 6:161250301 | G | A | 13.760 | 2.580 |
| ^*^ Weights for the association between the 43 genetic variants and lipoprotein(a) were acquired from Burgess et al. (2018)^1^. ^#^ Effect allele for rs139609547 was obtained from dbSNP by https://www.ncbi.nlm.nih.gov/snp/?term=rs139609547. | | | | | |
| 1. Burgess S, Ference BA, Staley JR, et al. Association of LPA Variants With Risk of Coronary Disease and the Implications for Lipoprotein(a)-Lowering Therapies: A Mendelian Randomization Analysis. JAMA Cardiol. 2018;3(7):619–627. doi:10.1001/jamacardio.2018.1470 | | | | | |

**Supplementary Table S2.** Definitions and sources of information for HF in the UK Biobank.

| **ICD-9** |
| --- |
| Congestive heart failure: 4280 |
| Left heart failure: 4281 |
| Heart failure, unspecified: 4289 |
| **ICD-10** |
| Hypertensive heart disease with (congestive) heart failure: I11.0 |
| Hypertensive heart and renal disease with (congestive) heart failure: I13.0 |
| Hypertensive heart and renal disease with both (congestive) heart failure and renal failure: I13.2 |
| Congestive heart failure: I50.0 |
| Left ventricular failure: I50.1 |
| Heart failure, unspecified: I50.9 |
| **Self-report^a^** |
| Non-cancer illness code: 20002 |

Abbreviation: HF, heart failure; ICD: International Classification of Disease.

^a^20002 is the data code used in UK Biobank: Non-cancer illness code.

**Supplementary Table S3.** Baseline participant characteristics by quintiles of Lp(a) PRS.

|  | **Lp(a) PRS Q1**  **(*n* =** **56,172)** | **Lp(a) PRS Q2**  **(*n* =** **56,171)** | **Lp(a) PRS Q3**  **(*n* =** **56,172)** | **Lp(a) PRS Q4**  **(*n* =** **56,191)** | **Lp(a) PRS Q5**  **(*n* =** **56,151)** |
| --- | --- | --- | --- | --- | --- |
| Lp(a) PRS | -4.3 (-140.8 to 0.1) | 6.2 (0.1 to 10.3) | 15.5 (10.3 to 22.9) | 36.5 (22.9 to 71.4) | 116.1 (71.4 to 388.4) |
| Lp(a), nmol/L | 8.1 (3.8 to 189.0) | 12.0 (3.8 to 188.9) | 17.3 (3.8 to 189.0) | 33.9 (3.8 to 189.0) | 121.3 (3.8 to 189.0) |
| Age, y | 58.0 (50.0-63.0) | 57.0 (50.0-63.0) | 57.0 (50.0-63.0) | 57.0 (50.0-63.0) | 57.0 (49.0-62.0) |
| Men, % | 40.3 | 42.5 | 42.7 | 42.6 | 45.1 |
| BMI, kg/m^2^ | 26.4 (23.9-29.4) | 26.5 (23.9-29.5) | 26.5 (23.9-29.5) | 26.5 (23.9-29.5) | 26.4 (23.8-29.6) |
| Current smoker, % | 9.9 | 9.8 | 9.9 | 9.9 | 9.8 |
| SBP, mmHg | 138.0 (125.0-152.0) | 138.0 (125.0-151.0) | 138.0 (125.0-152.0) | 138.0 (125.0-152.0) | 137.0 (125.0-151.0) |
| Antihypertensive medication use, % | 16.2 | 16.2 | 16.7 | 16.7 | 15.7 |
| Heart rate, bpm | 69.0 (62.0-76.0) | 68.0 (62.0-76.0) | 68.0 (62.0-76.0) | 69.0 (62.0-76.0) | 69.0 (62.0-76.0) |
| Diabetes, % | 4.1 | 4.2 | 4.4 | 4.3 | 4.5 |
| Parents had CVD, % | 40.4 | 40.2 | 40.6 | 40.9 | 42.5 |
| Siblings had CVD, % | 7.5 | 7.4 | 7.5 | 7.5 | 7.8 |
| Parents or siblings had CVD, % | 43.1 | 42.9 | 43.3 | 43.6 | 45.3 |

Continuous variables are described as median (interquartile range) and categorical variables are described as percentages.

Abbreviation: BMI, body mass index; CVD, cardiovascular disease; Lp(a), lipoprotein(a); SBP, systolic blood pressure.

Note: Circulating Lp(a) concentrations and Lp(a) PRS are described as median (range). Lp(a) PRS was estimated among the white only.

**Supplemental Table S4.** Baseline participant characteristics by parental and sibling history of CVD.

|  | **Parents and siblings did not have CVD**  **(*n* =170,315)** | **Parents or siblings had CVD**  **(*n* =128,843)** |
| --- | --- | --- |
| Age, y | 56.0 (48.0-62.0) | 58.0 (51.0-63.0) |
| Men, % | 45.1 | 39.4 |
| BMI, kg/m^2^ | 26.3 (23.8-29.4) | 26.6 (24.1-29.8) |
| Current smoker, % | 10.5 | 9.2 |
| SBP, mmHg | 136.0 (124.0-150.0) | 139.0 (126.0-153.0) |
| Antihypertensive medication use, % | 14.4 | 19.3 |
| Heart rate, bpm | 68.0 (62.0-76.0) | 69.0 (62.0-77.0) |
| Diabetes, % | 4.4 | 5.1 |
| Lp(a) PRS | 15.2 (3.0-49.2) | 15.9 (3.4-54.3) |
| Lp(a), nmol/L |  |  |
| White | 19.1 (9.1-55.9) | 21.6 (9.7-64.3) |
| Black or Black British | 68.0 (40.2-106.9) | 68.0 (42.5-106.3) |
| Asian or Asian British | 29.4 (12.6-61.1) | 32.7 (14.1-66.4) |
| Chinese | 16.3 (8.6-38.3) | 16.7 (9.2-43.5) |
| Mixed | 30.6 (12.1-65.0) | 31.2 (11.6-67.7) |
| Other | 34.6 (12.5-71.7) | 31.6 (11.9-67.2) |

Continuous variables are described as median (interquartile range) and categorical variables are described as percentages.

Abbreviation: BMI, body mass index; CVD, cardiovascular disease; Lp(a), Lipoprotein(a); PRS, polygenetic risk score; SBP, systolic blood pressure

**Supplemental Table S5.** Baseline participant characteristics by parental history of CVD.

|  | **Without a parental history of CVD**  **(*n* =** **178,535)** | **With a parental history of CVD**  **(*n* =** **120,623)** |
| --- | --- | --- |
| Age, y | 56.0 (48.0-62.0) | 58.0 (51.0-63.0) |
| Men, % | 44.9 | 39.3 |
| BMI, kg/m^2^ | 26.4 (23.9-29.4) | 26.6 (24.0-29.7) |
| Current smoker, % | 10.5 | 9.1 |
| SBP, mmHg | 137.0 (124.0-150.0) | 139.0 (126.0-152.0) |
| Antihypertensive medication use, % | 14.8 | 19.0 |
| Heart rate, bpm | 68.0 (62.0-76.0) | 69.0 (62.0-77.0) |
| Diabetes, % | 4.5 | 5.0 |
| Lp(a) PRS | 15.2 (3.0, 49.2) | 15.9 (3.4, 54.4) |
| Lp(a), nmol/L |  |  |
| White | 19.2 (9.1-56.3) | 21.6 (9.7-64.3) |
| Black or Black British | 68.0 (40.3-106.9) | 67.7 (41.1-105.7) |
| Asian or Asian British | 29.6 (12.7-61.4) | 32.8 (14.1-66.7) |
| Chinese | 16.0 (8.6-36.8) | 17.3 (9.5-45.0) |
| Mixed | 30.7 (12.0-64.6) | 30.8 (12.0-68.3) |
| Other | 34.5 (12.6-70.9) | 31.6 (11.6-68.0) |

Continuous variables are described as median (interquartile range), and categorical variables are described as percentages.

Abbreviation: BMI, body mass index; CVD, cardiovascular disease; Lp(a), Lipoprotein(a); PRS, polygenetic risk score; SBP, systolic blood pressure.

**Supplemental Table S6.** Baseline participant characteristics by sibling history of CVD.

|  | **Without a sibling history of CVD**  **(*n* =** **276,518)** | **With a sibling history of CVD**  **(*n* =** **22,640)** |
| --- | --- | --- |
| Age, y | 56.0 (49.0-62.0) | 61.0 (55.0-65.0) |
| Men, % | 43.1 | 37.4 |
| BMI, kg/m^2^ | 26.4 (23.9-29.5) | 27.1 (24.5-30.3) |
| Current smoker, % | 9.9 | 10.0 |
| SBP, mmHg | 137.0 (125.0-151.0) | 142.0 (129.0-156.0) |
| Antihypertensive medication use, % | 15.8 | 24.9 |
| Heart rate, bpm | 69.0 (62.0-76.0) | 69.0 (62.0-77.0) |
| Diabetes, % | 4.5 | 6.9 |
| Lp(a) PRS | 15.5 (3.1, 51.9) | 15.9 (3.2, 54.1) |
| Lp(a), nmol/L |  |  |
| White | 19.9 (9.3-58.9) | 22.4 (10.0-65.7) |
| Black or Black British | 67.9 (40.3-106.8) | 69.3 (44.5-104.4) |
| Asian or Asian British | 30.3 (13.0-62.9) | 35.5 (15.3-66.5) |
| Chinese | 16.5 (8.7-40.6) | 13.7 (10.0-31.4) |
| Mixed | 30.7 (12.1-66.7) | 33.1 (10.9-57.6) |
| Other | 33.4 (12.2-70.9) | 31.9 (12.3-62.2) |

Continuous variables are described as median (interquartile range), and categorical variables are described as percentages.

Abbreviation: BMI, body mass index; CVD, cardiovascular disease; Lp(a), Lipoprotein(a); PRS, polygenetic risk score; SBP, systolic blood pressure.

**Supplementary Table S7.** Hazard ratios for HF according to circulating ethnicity-specific quintiles of Lp(a), stratified by different types of FHx of CVD.

|  | **Events/person-y** | **Adjusted HR (95% CI)** |
| --- | --- | --- |
| **By parental history of CVD and sibling history of CVD** |  |  |
| **Parents and siblings did not have CVD** |  |  |
| Circulating ethnicity-specific Q1-Q4 | 2,183/1,599,613 | Ref. |
| Circulating ethnicity-specific Q5 | 549/374,966 | 1.10 (1.00, 1.21) |
| **Parents or siblings had CVD** |  |  |
| Circulating ethnicity-specific Q1-Q4 | 2,133/1,174,924 | Ref. |
| Circulating ethnicity-specific Q5 | 637/318,380 | 1.15 (1.06, 1.26) |
| **By parental history of CVD** |  |  |
| **Without a parental history of CVD** |  |  |
| Circulating ethnicity-specific Q1-Q4 | 2,383/1,674,109 | Ref. |
| Circulating ethnicity-specific Q5 | 619/394,581 | 1.13 (1.04, 1.24) |
| **With a parental history of CVD** |  |  |
| Circulating ethnicity-specific Q1-Q4 | 1,933/1,100,428 | Ref. |
| Circulating ethnicity-specific Q5 | 567/298,765 | 1.13 (1.03, 1.24) |
| **By sibling history of CVD** |  |  |
| **Without a sibling history of CVD** |  |  |
| Circulating ethnicity-specific Q1-Q4 | 3,764/2,570,197 | Ref. |
| Circulating ethnicity-specific Q5 | 1,025/637,644 | 1.13 (1.06, 1.21) |
| **With a sibling history of CVD** |  |  |
| Circulating ethnicity-specific Q1-Q4 | 552/204,340 | Ref. |
| Circulating ethnicity-specific Q5 | 161/55,702 | 1.14 (0.96, 1.36) |

HRs were adjusted for ARIC sans-BNP score, including age, sex, ethnicity, smoking status, BMI, SBP, antihypertensive medication use, heart rate, and diabetes

Abbreviation: CI, confidence interval; CVD, cardiovascular disease; HR, hazard ratios; HF, heart failure; FHx, family history; Lp(a), Lipoprotein(a).

**Supplementary Table S8.** Hazard ratios for HF according to quintiles of Lp(a) PRS, stratified by different types of FHx of CVD, among the white.

|  | **Events/person-y** | **Adjusted HR (95% CI)** |
| --- | --- | --- |
| **By parental history of CVD and sibling history of CVD** |  |  |
| **Parents and siblings did not have CVD** |  |  |
| Lp(a) PRS Q1-Q4 | 2,035/1,482,021 | Ref. |
| Lp(a) PRS Q5 | 516/356,990 | 1.10 (0.99, 1.21) |
| **Parents or siblings had CVD** |  |  |
| Lp(a) PRS Q1-Q4 | 2,062/1,125,882 | Ref. |
| Lp(a) PRS Q5 | 596/295,360 | 1.12 (1.02, 1.23) |
| **By parental history of CVD** |  |  |
| **Without a parental history of CVD** |  |  |
| Lp(a) PRS Q1-Q4 | 2,223/1,551,116 | Ref. |
| Lp(a) PRS Q5 | 575/375,080 | 1.11 (1.01, 1.22) |
| **With a parental history of CVD** |  |  |
| Lp(a) PRS Q1-Q4 | 1,874/1,056,787 | Ref. |
| Lp(a) PRS Q5 | 537/277,270 | 1.11 (1.01, 1.22) |
| **By sibling history of CVD** |  |  |
| **Without a sibling history of CVD** |  |  |
| Lp(a) PRS Q1-Q4 | 3,574/2,415,174 | Ref. |
| Lp(a) PRS Q5 | 963/601,708 | 1.11 (1.03, 1.19) |
| **With a sibling history of CVD** |  |  |
| Lp(a) PRS Q1-Q4 | 523/192,728 | Ref. |
| Lp(a) PRS Q5 | 149/50,642 | 1.09 (0.91, 1.31) |

HRs were adjusted for ARIC sans-BNP score, including age, sex, ethnicity, smoking status, BMI, SBP, antihypertensive medication use, heart rate, diabetes, and the first 10 principal components of ancestry.

Abbreviation: CI, confidence interval; CVD, cardiovascular disease; HR, hazard ratios; HF, heart failure; FHx, family history; Lp(a), Lipoprotein(a); PRS, polygenetic risk score.

**Supplementary Table S9.** Hazard ratios for HF according to circulating Lp(a) cut-off, stratified by different types of FHx of CVD.

|  | **Events/person-y** | **Adjusted HR (95% CI)** |
| --- | --- | --- |
| **By parental history of CVD and sibling history of CVD** |  |  |
| **Parents and siblings did not have CVD** |  |  |
| Circulating Lp(a) <50 mg/dL (105 nmol/L) | 2,325/1,693,077 | Ref. |
| Circulating Lp(a) ≥50 mg/dL (105 nmol/L) | 407/281,502 | 1.08 (0.97, 1.20) |
| **Parents or siblings had CVD** |  |  |
| Circulating Lp(a) <50 mg/dL (105 nmol/L) | 2,270/1,250,854 | Ref. |
| Circulating Lp(a) ≥50 mg/dL (105 nmol/L) | 500/242,450 | 1.19 (1.08, 1.31) |
| **By parental history of CVD** |  |  |
| **Without a parental history of CVD** |  |  |
| Circulating Lp(a) <50 mg/dL (105 nmol/L) | 2,538/1,771,923 | Ref. |
| Circulating Lp(a) ≥50 mg/dL (105 nmol/L) | 464/296,766 | 1.12 (1.01, 1.24) |
| **With a parental history of CVD** |  |  |
| Circulating Lp(a) <50 mg/dL (105 nmol/L) | 2,057/1,172,008 | Ref. |
| Circulating Lp(a) ≥50 mg/dL (105 nmol/L) | 443/227,185 | 1.15 (1.04, 1.28) |
| **By sibling history of CVD** |  |  |
| **Without a sibling history of CVD** |  |  |
| Circulating Lp(a) <50 mg/dL (105 nmol/L) | 4,010/2,726,835 | Ref. |
| Circulating Lp(a) ≥50 mg/dL (105 nmol/L) | 779/481,006 | 1.13 (1.04, 1.22) |
| **With a sibling history of CVD** |  |  |
| Circulating Lp(a) <50 mg/dL (105 nmol/L) | 585/217,096 | Ref. |
| Circulating Lp(a) ≥50 mg/dL (105 nmol/L) | 128/42,946 | 1.21 (1.00, 1.47) |

HRs were adjusted for ARIC sans-BNP score, including age, sex, ethnicity, smoking status, BMI, SBP, antihypertensive medication use, heart rate, and diabetes

Abbreviation: CI, confidence interval; CVD, cardiovascular disease; HF, heart failure; FHx, family history; HR, hazard ratios; Lp(a), Lipoprotein(a).

**Supplementary Table S10.** Subgroup analysis for the joint associations of circulating Lp(a) and FHx of CVD with risk of HF, stratified by different subgroups.

|  | **Parents or siblings had CVD** | | | | | | |
| --- | --- | --- | --- | --- | --- | --- | --- |
|  | **No** | |  | **Yes** | |  | ***P* for interaction** |
|  | **Circulating ethnicity-specific Q1-Q4** | **Circulating ethnicity-specific Q5** |  | **Circulating ethnicity-specific Q1-Q4** | **Circulating ethnicity-specific Q5** |  |  |
| **Age** |  |  |  |  |  |  | **0.935** |
| **< 60 y** |  |  |  |  |  |  |  |
| Events/person-y | 620/1,012,846 | 166/242,084 |  | 594/666,623 | 186/185,554 |  |  |
| HR (95% CI) | Ref. | 1.15 (0.97, 1.37) |  | 1.28 (1.14, 1.43) | 1.49 (1.26, 1.76) |  |  |
| **≥ 60 y** |  |  |  |  |  |  |  |
| Events/person-y | 1,563/586,767 | 383/132,881 |  | 1,539/508,300 | 451/132,826 |  |  |
| HR (95% CI) | Ref. | 1.08 (0.97, 1.21) |  | 1.15 (1.07, 1.23) | 1.32 (1.19, 1.47) |  |  |
| **Sex** |  |  |  |  |  |  | **0.083** |
| **Women** |  |  |  |  |  |  |  |
| Events/person-y | 849/885,279 | 213/204,258 |  | 981/715,237 | 280/194,800 |  |  |
| HR (95% CI) | Ref. | 1.11 (0.96, 1.29) |  | 1.16 (1.06, 1.28) | 1.26 (1.10, 1.44) |  |  |
| **Men** |  |  |  |  |  |  |  |
| Events/person-y | 1,334/714,334 | 336/170,707 |  | 1,152/459,686 | 357/123,580 |  |  |
| HR (95% CI) | Ref. | 1.09 (0.97, 1.23) |  | 1.19 (1.10, 1.28) | 1.45 (1.29, 1.63) |  |  |
| **Ethnicity** |  |  |  |  |  |  | **0.318** |
| **Non-white** |  |  |  |  |  |  |  |
| Events/person-y | 123/93,474 | 32/22,729 |  | 69/44,993 | 20/11,686 |  |  |
| HR (95% CI) | Ref. | 1.06 (0.72, 1.57) |  | 1.03 (0.77, 1.40) | 1.31 (0.81, 2.12) |  |  |
| **White** |  |  |  |  |  |  |  |
| Events/person-y | 2,060/1,506,139 | 517/352,236 |  | 2,064/1,129,931 | 617/306,694 |  |  |
| HR (95% CI) | Ref. | 1.10 (1.00, 1.21) |  | 1.19 (1.12, 1.26) | 1.37 (1.25, 1.50) |  |  |
| **BMI** |  |  |  |  |  |  | **0.144** |
| **<30 kg/m^2^** |  |  |  |  |  |  |  |
| Events/person-y | 1,362/1,264,094 | 346/295,416 |  | 1,301/899,869 | 384/243,930 |  |  |
| HR (95% CI) | Ref. | 1.10 (0.98, 1.24) |  | 1.20 (1.11, 1.30) | 1.35 (1.21, 1.52) |  |  |
| **≥30 kg/m^2^** |  |  |  |  |  |  |  |
| Events/person-y | 821/335,519 | 203/79,549 |  | 832/275,055 | 253/74,450 |  |  |
| HR (95% CI) | Ref. | 1.09 (0.94, 1.27) |  | 1.15 (1.04, 1.26) | 1.39 (1.20, 1.60) |  |  |
| **Ever smoke** |  |  |  |  |  |  | **0.601** |
| **No** |  |  |  |  |  |  |  |
| Events/person-y | 938/914,359 | 242/214,792 |  | 972/668,977 | 299/183,360 |  |  |
| HR (95% CI) | Ref. | 1.11 (0.96, 1.28) |  | 1.23 (1.12, 1.35) | 1.45 (1.27, 1.65) |  |  |
| **Yes** |  |  |  |  |  |  |  |
| Events/person-y | 1,245/685,253 | 307/160,173 |  | 1,161/505,946 | 338/135,020 |  |  |
| HR (95% CI) | Ref. | 1.09 (0.96, 1.23) |  | 1.14 (1.05, 1.24) | 1.30 (1.15, 1.47) |  |  |
| **Hypertension** |  |  |  |  |  |  | **0.849** |
| **No** |  |  |  |  |  |  |  |
| Events/person-y | 505/791,586 | 131/187,287 |  | 507/502,635 | 149/137,517 |  |  |
| HR (95% CI) | Ref. | 1.11 (0.91, 1.34) |  | 1.41 (1.24, 1.59) | 1.58 (1.32, 1.90) |  |  |
| **Yes** |  |  |  |  |  |  |  |
| Events/person-y | 1,678/808,027 | 418/187,678 |  | 1626/672,288 | 488/180,863 |  |  |
| HR (95% CI) | Ref. | 1.10 (0.99, 1.22) |  | 1.14 (1.06, 1.22) | 1.32 (1.20, 1.46) |  |  |
| **Diabetes** |  |  |  |  |  |  | **0.416** |
| **No** |  |  |  |  |  |  |  |
| Events/person-y | 1,874/1,531,366 | 476/359,316 |  | 1,828/1,117,033 | 547/303,621 |  |  |
| HR (95% CI) | Ref. | 1.11 (1.01, 1.23) |  | 1.21 (1.13, 1.29) | 1.38 (1.26, 1.52) |  |  |
| **Yes** |  |  |  |  |  |  |  |
| Events/person-y | 5,802/68,247 | 1,325/15,649 |  | 4,889/57,890 | 1,230/14,759 |  |  |
| HR (95% CI) | Ref. | 1.03 (0.80, 1.33) |  | 1.13 (0.97, 1.33) | 1.38 (1.09, 1.75) |  |  |

Adjustments were made for ARIC sans-BNP score, including age, sex, ethnicity, smoking status, BMI, SBP, antihypertensive medication use, heart rate, and diabetes.

Abbreviation: CI, confidence interval; CVD, cardiovascular disease; HR, hazard ratios; HF, heart failure; FHx, family history; Lp(a), Lipoprotein(a).

**Supplementary Table S11.** Subgroup analysis for the joint associations of Lp(a) PRS and FHx of CVD with risk of HF, stratified by different subgroups.

|  | **Parents or siblings had CVD** | | | | | | |
| --- | --- | --- | --- | --- | --- | --- | --- |
|  | **No** | |  | **Yes** | |  | ***P* for interaction** |
|  | **Lp(a) PRS Q1-Q4** | **Lp(a) PRS Q5** |  | **Lp(a) PRS Q1-Q4** | **Lp(a) PRS Q5** |  |  |
| **Age** |  |  |  |  |  |  | **0.397** |
| **< 60 y** |  |  |  |  |  |  |  |
| Events/person-y | 544/915,388 | 162/232,487 |  | 546/627,080 | 180/172,329 |  |  |
| HR (95% CI) | Ref. | 1.18 (0.99, 1.41) |  | 1.28 (1.14, 1.44) | 1.54 (1.30, 1.82) |  |  |
| **≥ 60 y** |  |  |  |  |  |  |  |
| Events/person-y | 1,491/566,632 | 354/124,503 |  | 1,516/498,801 | 416/123,031 |  |  |
| HR (95% CI) | Ref. | 1.05 (0.94, 1.18) |  | 1.16 (1.08, 1.25) | 1.28 (1.15, 1.43) |  |  |
| **Sex** |  |  |  |  |  |  | **0.157** |
| **Women** |  |  |  |  |  |  |  |
| Events/person-y | 805/827,001 | 188/187,310 |  | 962/693,602 | 240/172,955 |  |  |
| HR (95% CI) | Ref. | 1.14 (0.97, 1.33) |  | 1.17 (1.06, 1.28) | 1.25 (1.08, 1.44) |  |  |
| **Men** |  |  |  |  |  |  |  |
| Events/person-y | 1,230/655,019 | 328/169,680 |  | 1,100/432,280 | 356/122,405 |  |  |
| HR (95% CI) | Ref. | 1.06 (0.94, 1.20) |  | 1.21 (1.11, 1.31) | 1.42 (1.26, 1.59) |  |  |
| **BMI** |  |  |  |  |  |  | **0.501** |
| **<30 kg/m^2^** |  |  |  |  |  |  |  |
| Events/person-y | 1,282/1,177,209 | 322/281,149 |  | 1,260/863,658 | 360/225,990 |  |  |
| HR (95% CI) | Ref. | 1.10 (0.97, 1.24) |  | 1.20 (1.11, 1.30) | 1.36 (1.21, 1.53) |  |  |
| **≥30 kg/m^2^** |  |  |  |  |  |  |  |
| Events/person-y | 753/304,811 | 194/75,840 |  | 802/262,223 | 236/69,370 |  |  |
| HR (95% CI) | Ref. | 1.06 (0.91, 1.24) |  | 1.17 (1.06, 1.30) | 1.31 (1.13, 1.52) |  |  |
| **Ever smoke** |  |  |  |  |  |  | **0.487** |
| **No** |  |  |  |  |  |  |  |
| Events/person-y | 851/833,297 | 221/203,360 |  | 944/635,237 | 259/168,202 |  |  |
| HR (95% CI) | Ref. | 1.09 (0.94, 1.27) |  | 1.27 (1.15, 1.39) | 1.34 (1.17, 1.54) |  |  |
| **Yes** |  |  |  |  |  |  |  |
| Events/person-y | 1,184/648,724 | 295/153,630 |  | 1,118/490,645 | 337/127,158 |  |  |
| HR (95% CI) | Ref. | 1.08 (0.95, 1.23) |  | 1.13 (1.04, 1.23) | 1.35 (1.20, 1.52) |  |  |
| **Hypertension** |  |  |  |  |  |  | **0.748** |
| **No** |  |  |  |  |  |  |  |
| Events/person-y | 465/729,800 | 132/180,105 |  | 488/478,056 | 141/127,684 |  |  |
| HR (95% CI) | Ref. | 1.18 (0.98, 1.44) |  | 1.42 (1.25, 1.61) | 1.62 (1.34, 1.96) |  |  |
| **Yes** |  |  |  |  |  |  |  |
| Events/person-y | 1,570/752,220 | 384/176,884 |  | 1,574/647,826 | 455/167,676 |  |  |
| HR (95% CI) | Ref. | 1.06 (0.94, 1.18) |  | 1.14 (1.07, 1.23) | 1.29 (1.16, 1.43) |  |  |
| **Diabetes** |  |  |  |  |  |  | **0.450** |
| **No** |  |  |  |  |  |  |  |
| Events/person-y | 1,781/1,426,202 | 452/342,787 |  | 1,792/1,075,227 | 507/281,480 |  |  |
| HR (95% CI) | Ref. | 1.10 (0.99, 1.22) |  | 1.21 (1.14, 1.30) | 1.35 (1.23, 1.49) |  |  |
| **Yes** |  |  |  |  |  |  |  |
| Events/person-y | 254/55,818 | 64/14,203 |  | 270/50,655 | 89/13,880 |  |  |
| HR (95% CI) | Ref. | 1.00 (0.76, 1.32) |  | 1.17 (0.98, 1.39) | 1.41 (1.11, 1.80) |  |  |

Adjustments were made for ARIC sans-BNP score, including age, sex, ethnicity, smoking status, BMI, SBP, antihypertensive medication use, heart rate, diabetes, and the first 10 principal components of ancestry.

Abbreviation: CI, confidence interval; CVD, cardiovascular disease; HR, hazard ratios; HF, heart failure; FHx, family history; Lp(a), Lipoprotein(a); PRS, polygenetic risk score

**Supplementary Table S12.** Improvement in HF risk reclassification with Lp(a) and different types of family history of CVD.

|  | **Circulating Lp(a) & FHx** |  | **Lp(a) PRS & FHx** |
| --- | --- | --- | --- |
|  | **NRI (95% CI)** |  | **NRI (95% CI)** |
| Lp(a) | 0.0471 (0.0211, 0.0734) |  | 0.0337 (0.0047, 0.0656) |
| Parents had CVD | 0.1024 (0.0698, 0.1300) |  | 0.1109 (0.0759, 0.1425) |
| Lp(a) + parents had CVD | 0.1037 (0.0721, 0.1341) |  | 0.1106 (0.0784, 0.1429) |
| Siblings had CVD | 0.0405 (0.0214, 0.0654) |  | 0.0392 (0.0168, 0.0641) |
| Lp(a) + siblings had CVD | 0.0946 (0.0658, 0.1207) |  | 0.0765 (0.0464, 0.1122) |
| Parents or siblings had CVD | 0.1504 (0.1146, 0.1810) |  | 0.1566 (0.1197, 0.1910) |
| Lp(a) + parents or siblings had CVD | 0.1576 (0.1069, 0.1976) |  | 0.1638 (0.1130, 0.2030) |

Change in risk reclassification after adding Lp(a) and different types of family history of CVD individually and in combination to a baseline risk prediction model comprising ARIC sans-BNP score, including age, sex, ethnicity, smoking status, BMI, SBP, antihypertensive medication use, heart rate, and diabetes.

Abbreviation: CVD, cardiovascular disease; Lp(a), Lipoprotein(a); NRI, net reclassification improvement; PRS, polygenetic risk score; SBP, systolic blood pressure

**Supplementary Figure S1.** Hazard ratios for the joint associations of predefined Lp(a) cut-off and different types of FHx of CVD with incident HF.


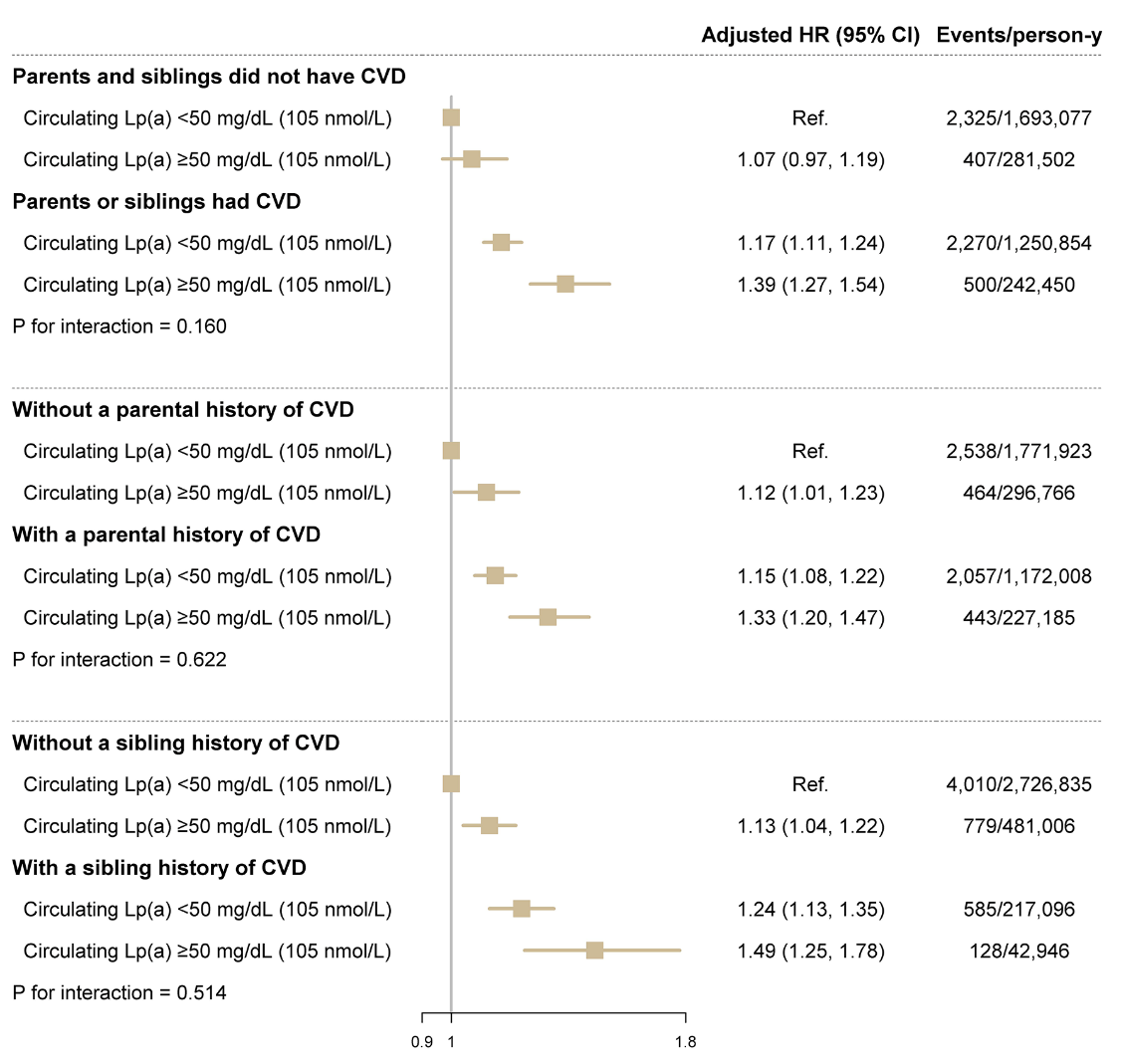


HRs were adjusted for ARIC sans-BNP score, including age, sex, ethnicity, smoking status, BMI, SBP, antihypertensive medication use, heart rate, and diabetes.

Abbreviation: CI, confidence interval; HR, hazard ratios; HF, heart failure; FHx, family history; Lp(a), Lipoprotein(a); CVD, cardiovascular disease
